# Uncovering Tumour Heterogeneity through PKR and nc886 Analysis in Metastatic Colon Cancer Patients Treated with 5-FU-Based Chemotherapy

**DOI:** 10.3390/cancers12020379

**Published:** 2020-02-07

**Authors:** María Belén Ortega-García, Alberto Mesa, Elisa L.J. Moya, Beatriz Rueda, Gabriel Lopez-Ordoño, Javier Ángel García, Verónica Conde, Eduardo Redondo-Cerezo, Javier Luis Lopez-Hidalgo, Gema Jiménez, Macarena Peran, Luis J. Martínez-González, Coral del Val, Igor Zwir, Juan Antonio Marchal, María Ángel García

**Affiliations:** 1 Instituto de Investigación Biosanitaria ibs.GRANADA, 18012 Granada, Spain; 2 Department of Oncology, Virgen de las Nieves University Hospital, 18014 Granada, Spain; 3 Biopathology and Regenerative Medicine Institute (IBIMER), Centre for Biomedical Research, (CIBM) University of Granada, 18100 Granada, Spain; 4 Excellence Research Unit “Modelling Nature” (MNat), University of Granada, 18071 Granada, Spain; 5 Andalusian Research Institute in Data Science and Computational Intelligence (DaSCI Institute), 18014 Granada, Spain; 6 Department of Pathology, San Cecilio University Hospital, 18016 Granada, Spain; 7 Department of Gastroenterology, Torrecardenas Hospital, 04009 Almería, Spain; 8 Department of Gastroenterology, Virgen de las Nieves University Hospital, 18014 Granada, Spain; 9 Department of Health Sciences, University of Jaén, 23071 Jaen, Spain; 10 GENYO: Centre for Genomics and Oncological Research: Pfizer/University of Granada/Andalusian Regional Government, PTS, 18007 Granada, Spain; 11 Department of Computer Science and Artificial Intelligence, University of Granada, 18071 Granada, Spain; 12 Department of Psychiatry, Washington University School of Medicine, St Louis, MO 63110, USA; 13 Department of Human Anatomy and Embryology, University of Granada, 18016 Granada, Spain; 14 Department of Biochemistry and Molecular Biology III, University of Granada, 18016 Granada, Spain

**Keywords:** colorectal cancer, 5-fluorouracil-based chemotherapy, protein kinase PKR, non-coding nc886, ambispective study, cluster of patients, biomarkers

## Abstract

Colorectal cancer treatment has advanced over the past decade. The drug 5-fluorouracil is still used with a wide percentage of patients who do not respond. Therefore, a challenge is the identification of predictive biomarkers. The protein kinase R (PKR also called EIF2AK2) and its regulator, the non-coding pre-mir-nc886, have multiple effects on cells in response to numerous types of stress, including chemotherapy. In this work, we performed an ambispective study with 197 metastatic colon cancer patients with unresectable metastases to determine the relative expression levels of both nc886 and PKR by qPCR, as well as the location of PKR by immunohistochemistry in tumour samples and healthy tissues (plasma and colon epithelium). As primary end point, the expression levels were related to the objective response to first-line chemotherapy following the response evaluation criteria in solid tumours (RECIST) and, as the second end point, with survival at 18 and 36 months. Hierarchical agglomerative clustering was performed to accommodate the heterogeneity and complexity of oncological patients’ data. High expression levels of nc886 were related to the response to treatment and allowed to identify clusters of patients. Although the PKR mRNA expression was not associated with chemotherapy response, the absence of PKR location in the nucleolus was correlated with first-line chemotherapy response. Moreover, a relationship between survival and the expression of both PKR and nc886 in healthy tissues was found. Therefore, this work evaluated the best way to analyse the potential biomarkers PKR and nc886 in order to establish clusters of patients depending on the cancer outcomes using algorithms for complex and heterogeneous data.

## 1. Introduction

Colorectal cancer (CRC) is one of the most common forms of cancer worldwide, being the third most commonly diagnosed malignancy and the second leading cause of cancer death in recent years [1]. Although CRC treatment has advanced over the past decades, treatment outcomes depend, in part, on tumour- and patient-specific molecular characteristics [2]. Even though many novel drugs have been developed for patients with advanced CRC, 5-fluorouracil (5-FU) is still widely used as the classic and basic drug in adjuvant chemotherapy and palliative care. 5-FU is used as an infusion, taken orally (capecitabine), or used in combination with different drugs (FOLFOX, FOLFIRI) but its efficacy is limited by numerous factors including tumour cell genetics, epigenetics, and proteomics, which promote chemoresistance and metastasis [2]. In the last decade, the efficacy of these regimens has been increased by incorporating new biological therapies based on the use of monoclonal antibodies [3]. Despite the fact of the considerable improvement in the efficacy, there are still a wide percentage of patients who do not benefit from 5-FU-based treatments. Therefore, the identification of biomarkers that associate or predict the benefit of an appropriate selection of candidates for 5-FU-based therapies and combined therapies constitutes a broad area in clinical and translational research. 

The protein kinase R (PKR, also called EIF2AK2) is an interferon-inducible double-stranded RNA protein kinase with multiple effects on cells. This protein kinase plays an active part in the cellular response to numerous types of stress mediating in several biological pathways and with a potent role in the induction of apoptosis in response to numerous compounds [4]. PKR is a serine-threonine kinase, composed by the kinase domain shared by the other eukaryotic initiation factor 2 alpha (eIF2α) kinases, and two dsRNA binding domains (dsRBD) that regulate its activity. PKR autophosphorylation represents the activation reaction that leads to the phosphorylation of eIF-2α, impairing eIF-2 activity, which results in the inhibition of protein synthesis [5]. In addition to its translational regulatory function, PKR has a role in signal transduction and transcriptional control through the IκB inhibitor/ nuclear transcription factor NF-κB pathway [6]. Although the primary PKR activator is dsRNA—produced during infection by several viruses and detected at low levels in mammalian cells—PKR is also activated by a variety of cellular stresses including cytokines, calcium stress, oxidative stress, endoplasmic reticulum stress, lipo-stress, amyloid-β (Aβ) peptide accumulation, polyanions such as heparin, and several drugs, or through the PKR-associated activator (PACT) [7]. PKR, which is expressed constitutively in mammalian cells, has also been implicated in the control of cell growth and differentiation with debated antitumor role and as an important antiviral agent [7]. Recently, the role of PKR related to metabolism, inflammatory processes, cancer, and neurodegenerative diseases has gained great interest [7,8].

The importance of PKR function in cell growth, differentiation, stress response, and immunomodulation is further noted by the existence of numerous modulators. Therefore, it has been identified that several PKR regulators are involved in cancer outcome, where the non-coding RNA pre-miR-886, also called nc886 or vault vtRNA 2-1, has been described as a potent regulator of PKR [9,10,11]. Nc886 binds to PKR with an affinity comparable to dsRNA and prevents PKR from being activated, in contrast to the PKR-activating ligand dsRNA [9,10,11]. Although nc886 was initially discovered as a PKR inhibitor, recently researchers have demonstrated that nc886 can adopt two structurally distinct conformers that are functionally opposing regulators of PKR [12,13].

We have previously identified PKR as a molecular target of 5-FU in several colon and breast cancer cells lines, playing an important role in the cytotoxic effect of 5-FU at least, in part, through the induction of cell death by apoptosis [14]. Because PKR has also been implicated in the anti-tumour activity of chemotherapeutic drugs such as doxorubicin (DOX) and etoposide [15,16], and nc886 has been identified as an interesting tumour suppressor [17,18,19], we consider the analysis of PKR and the nc886 in patients as potential predictive biomarkers to be of clinical importance. For this reason, the aim of this work was to carry out an ambispective study in 197 metastatic colon cancer patients to evaluate the expression levels of PKR and its pre-microRNA-nc886 by qPCR in colon tumour samples and their respective healthy tissues and plasma, analysing its relation with the patient’s clinical evolution. The primary end point was the evaluation of these variables with the objective response (OR) to first-line of 5-FU-based chemotherapy determined by the response evaluation criteria in solid tumours (RECIST). As the second end point, we also analysed the relationship of these variables with overall survival (OS) at 18 and 36 months in those patients when the information was available. In addition, we analysed the PKR location by immunohistochemistry in 76 colon tumours and its respective colon healthy tissues. For all of this study, novel bioinformatic analyses have been included in order to distinguish parameters and signals for the identification of different profiles in patients, who have the same diagnostic and disease. Hence, clustering analyses were done using hierarchical agglomerative clustering (Statistical Toolbox, Matlab 2007, Spotfire Decision Site 9.1.2) [20], with the objective of improving the quality and specificity of the results considering the heterogeneity between samples and the genetic and proteomic background of oncologic patients.

## 2. Materials and Methods

### 2.1. Patients and Samples

The study was approved by the Biomedical Research Ethics Committee of Granada (Cod Peiba. 0170-N-16) and informed patient consent was obtained. The process of recruitment, traceability of samples, and informed consent was regulated and controlled by the Andalusian Public Health System Biobank (BBSSPA), according to the World Medical Association Declaration of Helsinki.

The study included a total of 197 colon metastatic cancer patients with unresectable lung or liver metastases from September 2014 through to September 2018 who were treated with 5-FU-based therapy as first-line treatment using the standard treatment schedule. To avoid discarding as few samples as possible, the missing values were approximated to the median value established for each variable analysed (see the supplementary Excel document with available data in Figure S1). Criteria of the RECIST guidelines were used to characterize the response to this treatment [21,22]. According to these criteria, after the first restaging assessment that was generally performed around 3–4 months after the initiation of 5-FU-based treatment, patients with progressive disease were considered as non-responders with primary resistance and those patients with partial response or stable disease under the 5-FU treatment for at least 3–4 months were considered as responders. Survival was considered after 18 months and 36 months in those patients where the data were available (see the supplementary Excel document with available data in Figure S1).

Tumours were classified according to the 2002 tumour, node, metastasis (TNM) classification and the Fuhrman grading system by experienced pathologists (B.R. and J.L.L.-H.) [23]. Archived formalin-fixed paraffin-embedded (FFPE) tissue samples from colon tumour and their corresponding surrounding healthy colon tissues obtained for routine diagnostic purposes were used in this study.

**Figure 1:**
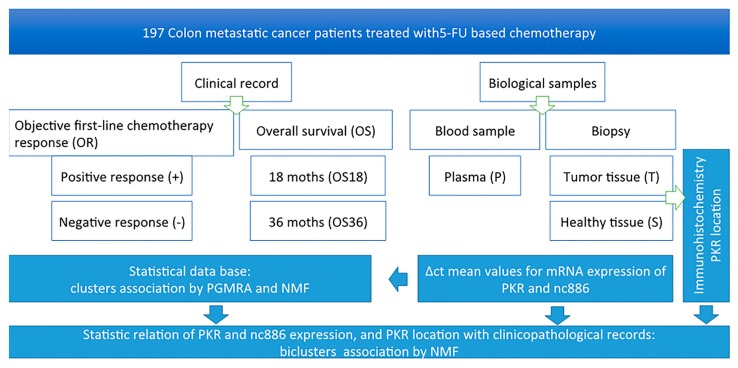
Flowchart of the ambispective study.

Peripheral blood samples of subjects were collected prospectively with one tube for EDTA anticoagulant (3 mL). Samples were centrifuged at 3000 rpm for 10 min and, then, aliquoted and frozen at −80 °C until use. The flow diagram shown in Figure 1 outlines the steps performed in this ambispective study. 

### 2.2. RNA Extraction from FFPE Tissue and from Plasma Sample

Haematoxylin and eosin-stained histological FFPE sections were prepared to identify areas of normal and tumour tissue. These regions of interest were biopsied by macrodissection after the xylene-alcohol dewaxing performed by specialist pathologists (Atrys Health S.A., Gr, SP). The tissue of interest was scraped with a scalpel and dipped in lysis buffer (ATL) (Qiagen, Hi, GE). Only tumour samples with more than 80% cancer cells were considered for further analysis. Non-malignant tissue with a distance of >100 mm to the cancer tissue was collected from all patients of the study cohort for comparison purposes. Total RNA was isolated from the dissected FFPE tissue samples using an miRNeasy FFPE Kit (Qiagen, Hi, Ge) and from plasma using the miRNeasy Serum-Plasma Kit (Qiagen, Hi, Ge) following the recommended protocol by previous concentration of 300 μL of cold plasma using the Vacufuge Concentrator system (Eppendorf, GE). Both RNA isolations were automated using the QIAcube (Qiagen, Hi, GE). Integrity and quality of RNA (RIN) was tested with Bioanalyzer (Agilent, CA) and diluted to a maximum concentration of 500 ng in 14 μL. The reverse transcription was performed with the SuperScript VILO cDNA Synthesis Kit (Invitrogen-Thermo Fisher Scientific, USA) on all samples (4 μL 5X VILO Reaction Mix, 2 μL 10X SuperScript Enzyme Mix, 14 μL RNA, 10 min at 25 °C, 60 min at 42 °C, and 5 min at 85 °C).

### 2.3. RT qPCR Assay

The determination of the expression of PKR gene and nc886 element was carried out in plasma, tumours, and healthy colon tissues from patients enrolled in this ambispective study. To determine the expression of the PKR (EIF2AK2) gene and nc886 (VTRNA2) element, specific fluorescent hydrolysis probes TaqMan-MGB (Thermo Fisher Scientific, MA, USA) were used (Hs01091582_m1, Hs04273370_s1, respectively) by real-time qPCR and by digital dPCR in different samples (Figures S2 and S3). To select the most appropriate endogenous control, we analysed the endogenous classic Glyceraldehyde 3-phosphate dehydrogenase (GADPH), Hypoxanthine-guanine phosphoribosyltransferase (HPRT), and β2 microglobulin (B2M) genes [24] by several tests to assess their stability in different types of tissue by qPCR (Figure S4). Due to the results obtained, B2M (Hs00187842_m1) was considered as the endogenous control for plasma and FFPE tissue samples. For the amplification of the samples, the “TaqMan Gene Expression Master Mix” protocol was adapted to a final volume of 10 μL in the QuantStudio 12K Flex system (Thermo Fisher Scientific, MA, USA). The mean Ct-values were technically normalized using the endogenous B2M, and the expression level was considered as 2^−ΔCt^ (ΔCt = Ct_targetgene_ − Ct_B2M_) [25,26,27]. The missing values were approximated to the median value established for each variable analysed (see the supplementary Excel document in Figure S1).

### 2.4. Immunohistochemistry Analysis

Formalin-fixed paraffin-embedded samples (*n* = 76) were cut at 2.5 μm in thickness and placed on a slide. The antigenic retrieval was carried out by incubating the antibody for 30 min with hydrogen peroxide (H_2_O_2_) at pH 8. The immunohistochemical technique was carried out on the Lab Vision Autostainer 480S (Thermo Fisher Scientific, MA, USA). For the development of the technique, the Commercial Kit Detection System Master Polymer Plus (Peroxidase) was used. The polyclonal anti-PKR antibody was administered by Santa Cruz Biotechnology, and it was used with a 1:50 dilution in 30 min of incubation. The development of the technique was carried out with diaminobenzidine (DAB) and after, with hematoxylin and eosin staining. The immunohistochemical location of the PKR protein was determined by two pathologists that considered the presence of the PKR protein in the nucleolus or outside of nucleolus (mostly located in cytoplasm).

### 2.5. Machine Learning and Statistical Analysis

PGMRA is a deep unsupervised [28,29] and data-driven machine learning method that combines model-based, consensus, fuzzy, possibilistic, relational, optimization, and conceptual clustering techniques into a single method (see the supplementary material in [30] for a review, [20,31]). The model-based approach uses non-negative matrix factorization to identify candidates for functional clusters [20,32] represented as tensors or flattened biclusters (e.g., subjects × symptoms). Biclusters can be learned independently of the number of clusters, and thus, from different granularity partitions (consensus). The method separately searches for biclusters in distinct domains of knowledge (e.g., genetics, clinical symptoms) without regard for their calculations in other domains of knowledge [33]. Then, the approach agnostically co-clusters the inter-domain biclusters and identifies natural relationships (associations) among them. Associations result from optimizing the probability of the intersection among biclusters using hypergeometric statistics or Fisher’s exact test [34,35] evaluated by a posterior permutation test instead of using typical inter/intra clustering metrics among dots in the n-dimensional space (model-based). Biclusters in one domain of knowledge or associations of biclusters from different domains of knowledge can be reorganized into networks at different levels of granularity, connected by sharing observations (subjects) and/or features (∆ct mean values, objective first-line chemotherapy response). This framework constitutes a knowledge base and characterizes architecture of the disease. The methodological basis of PGMRA is available in [20,31,34,35,36], and its web server application is online at http://phop.ugr.es/fenogeno [20]. Fast parallel software implementations were run at the Centre for High Performance Computing (CHPC) facility at Washington University School of Medicine (WUSM).

### 2.6. Derivation of the Empirical Index

First, we calculated a purely empirical (i.e., agnostic and data-driven) indicator of character functioning. We clustered subjects corresponding to the two expression variables and assigned each subject the number of the cluster to which they belonged (as described in the next paragraph). The result was a single empirical index of cluster membership that served as a comprehensive measure of variability in the RNA expression. 

To calculate the cluster rankings, we applied hierarchical agglomerative clustering (Statistical Toolbox, Matlab 2007b) [20] with a complete linkage method and correlation similarity measurement to group value phenotypic or environmental sets by their shared subjects using hypergeometric statistics. The function that controls the vertical order in which a row is plotted (Spotfire Decision Site 9.1.2) in a hierarchical clustering is defined as follows.

Given two sub-clusters within a cluster (there are always exactly two sub-clusters considered at each step), both sub-clusters are weighted and the sub-cluster with the highest weight is placed above the other sub-cluster. This function is systematically applied until a single cluster containing all rows is obtained. To calculate the weight *w*_3_ of a new cluster, *C*_3_ is formed from two sub-clusters *C*_1_ and *C*_2_ with a weight of *w*_1_ and *w*_2_, and each containing *n*_1_ and *n*_2_ rows, and the following expression is used:

(1) $$w3=\;\frac{n_{1}\;\;x\;\;w_{1}+n_{2}\;\;x\;\;w_{2}}{\left(n_{1}+n_{2}\right)}$$

The weight of a sub-cluster with a single row is calculated as the average value of its columns. 

### 2.7. Feature Selection Process Using Non-Negative Matrix Factorization (NMF) in PGMRA

We use non-negative matrix factorization (NMF) method as a deep autoencoder [29] in a particular domain of knowledge (qPCR data, clinical data) to identify candidates for functional clusters [20,32], represented as tensors or flattened biclusters (e.g., unknown relationships embedded in the data (subjects × non-coding RNA differential expression)). Our implementation of the NMF, termed fuzzy NMF method (FNMF), learns [20,30] biclusters independently of the number of clusters, and thus, from different granularity partitions (consensus). The method separately searches for biclusters in distinct domains of knowledge without regard for their calculations in other domain of knowledge [33]. Then, the approach agnostically co-clusters the inter-domain biclusters and identifies natural relationships (associations) among them. Associations result from optimizing the probability of the intersection among biclusters using hypergeometric statistics instead of using typical inter/intra clustering metrics among dots in the ndimensional space (model-based). These associations are learned regardless of any status of the observations (e.g., cases and controls, unsupervised) and are optimized on the basis of multiobjective and multimodal optimization techniques [37]. By incorporating a posteriori, a “supervised” status, the method is able to calculate the risk of the association by the frequency of one status vs. another. Once it occurs, the method becomes semi-supervised, and posterior statistical significance of the association is calculated using kernel-based and multivariate statistical analyses [20,38]. 

## 3. Results

### 3.1. Normalized Values of Non Coding nc886 in Plasma and Tumor Tissues Predicted the Objective First-Line Chemotherapy Response

We tested first the association of the expression level of the PKR gene and the nc886 RNA molecule determined by the ∆ct mean values identified by qPCR in tumour (T), plasma (P), and healthy (S) tissues (see the supplementary Excel document in Figure S1) with the OR to first-line chemotherapy. The response was encoded as a Boolean (positive/responders, negative/non-responders) tested after 3–4 months of starting the treatment as indicated in the Material and Methods section. 

**Figure 2:**
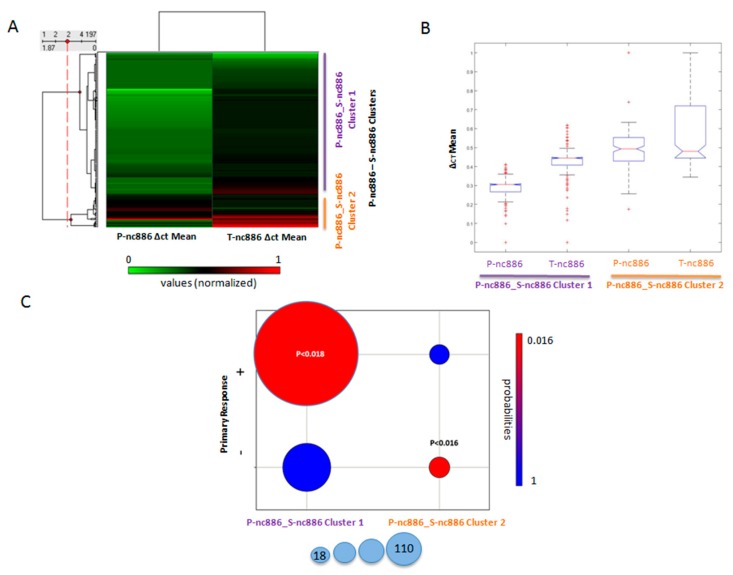
Association of the expression level of protein kinase R (PKR) and nc886 determined by the ∆ct mean values identified by RT qPCR in tumour (T), plasma (P), and healthy (S) tissues with the objective response to first-line chemotherapy. (**A**) P-T_nc886 clusters: Biclusters of subjects sharing ∆ct mean values of nc886 in P and T. ∆ct mean values are normalized between 0 (green) and 1 (red). (**B**) Boxplot of ∆ct mean values of nc886 in P and T for each cluster. (**C**) Correlation between the objective first-line chemotherapy response and the nc886 ∆ct mean clusters (Figure 2A). *p*-values were calculated with hypergeometric statistics. Colour code for *p*-values’ statistical significance is indicated from high (red) to low (blue). The size of the circles indicates the number of individuals in the relationship.

We identified two biclusters (subjects sharing subsets of features) composed of subjects sharing P-nc886 ∆ct mean and T-nc886 ∆ct mean values (Figure 2A). These biclusters exhibited significantly different values of their composite features (Figure 2B, *p* < 2.44655 × 10^−77^ ANOVA statistics). 

Those subjects were significantly associated with the objective first-line chemotherapy response (Figure 2C). The first bicluster displayed low P-nc886 and mid/low T-nc886 ∆ct mean values and was associated with a positive response (Figure 2C, *p* < 0.018, hypergeometric statistics/Fisher’s tests). The second bicluster exhibited high T-nc886 ∆ct mean values, and was associated with a negative response (Figure 2C, *p* < 0.016, hypergeometric statistics/Fisher’s tests). The other studied variables that involved the expression of PKR in the different samples were not included in any bicluster significantly associated with the objective first-line chemotherapy response (see non-negative matrix factorization (NMF) in PGMRA as a feature selection process in the Materials and Methods section). 

Once we detected the two highly associated variables, we independently validated the former results by performing a regression analysis between the cluster order (ranking, see the Materials and Methods section) and the individual features with respect to the OR to first-line chemotherapy. ∆ct values were normalized between [0,1] due to PGMRA requirements. We determined that the clusters, represented by the order of their observations, were better associated with the objective first-line chemotherapy response (*p* < 0.00012, F statistics) than the individual T-nc886 ∆ct mean values (*p* < 0.0028) and P-nc886 ∆ct mean values (*p* < 0.013) (data not shown). Moreover, all other variables involved in similar regressions were non-significantly associated with the OR. 

Furthermore, because the relative level of gene expression is inversely proportional to the ∆ct mean value following the 2^-ΔCt^ method [26], our data suggest for cluster 1 a significant association between patients who showed high level of expression of nc886 in both plasma and tumour samples with a significant positive response to treatments based on the use of 5-FU. In contrast, cluster 2 included patients who mostly and significantly showed lower levels of nc886 expression in the tumour and a negative response to treatment. However, the levels of expression of the PKR gene were not related to the OR to first-line chemotherapy. 

### 3.2. PKR Location Predicted the Objective First-Line Chemotherapy Response

**Figure 3:**
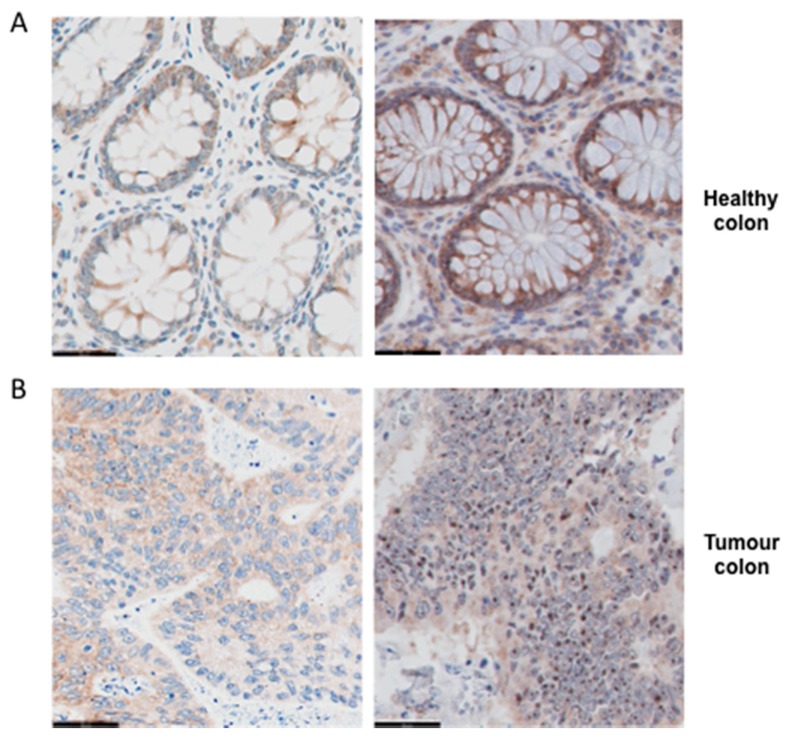
Different locations of PKR in healthy colon tissues and tumour tissues. (**A**) Representative immunohistochemical detection of total PKR in healthy colon tissues. PKR immunostaining was weak (first panel) or strong (second panel), but was mostly located in the cytoplasm of cells. Scale bar, 50 µm. (**B**) Representative immunohistochemical detection of total PKR in tumour colon tissues. PKR immunostaining was mostly located in the cytoplasm of cells (first panel); however, in several tumours, PKR was located heavily in the nucleolus (second panel). Scale bar, 50 µm.

Because the relative levels of expression of the PKR gene mRNA in the colon tumour could not be related to the patient’s response to the treatment, we decided to analyse the location of PKR in the tumour and healthy colon tissue cells by immuhistochemistry (*n* = 76). Although PKR was located in all healthy tissues analysed at the level of the cytoplasm of the cells, in tumour samples its location could be restricted to the nucleolus in some cases (Figure 3). Therefore, we considered the two variables of presence or absence (located in the cytoplasm) of PKR in the nucleolus. 

**Figure 4:**
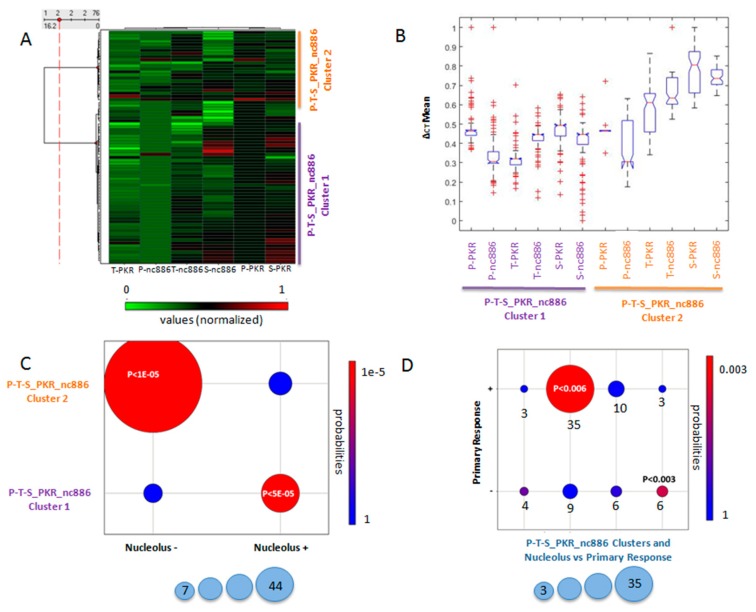
Association of the PKR location with ∆ct mean values identified by RT qPCR in tumor (T), plasma (P), and healthy (S) tissues and the objective response to first-line chemotherapy. (**A**) Clusters P-T-S_PKR_nc886: Biclusters of subjects sharing ∆ct mean values of PKR determined in the analysed tissues (P-PKR, T-PKR, S-PKR) and ∆ct mean values of nc886 determined in the analysed tissues (T-nc886, P-nc886, S-nc886). ∆ct Mean values are normalized between 0 (green) and 1 (red). (**B**) Boxplot of ∆ct mean values of PKR determined in the analysed tissues (P-PKR, T-PKR, S-PKR) and ∆ct mean values of nc886 determined in the analysed tissues (T-nc886, P-nc886, S-nc886) for each cluster P-T-S_PKR_nc886. (**C**) Co-clustering between the PKR location in the nucleolus and the clusters P-T-S_PKR_nc886. *p*-values were calculated with hypergeometric statistics. Colour code for *p*-values’ statistical significance is indicated from high (red) to low (blue). The size of the circles indicates the number of individuals in the relationship. (**D**) Co-clustering occurred between the objective first-line chemotherapy response and the co-clusters identified in (**C**). *p*-values were calculated with hypergeometric statistics. Colour code for *p*-values’ statistical significance is indicated from high (red) to low (blue). The size of the circles indicates the number of individuals in the relationship.

To test the predictive value of the PKR location, we analysed the patients for which this information was available. Two biclusters were obtained by PGMRA when including PKR ∆ct mean values in P, T, and S (P-PKR, T-PKR, S-PKR) and the ∆ct mean values of nc886 in P, T, and S (T-nc886, P-nc886, S-nc886) (Figure 4A). The first bicluster displayed variable ∆ct mean values (high S-PKR, medium P-PKR, low or medium T-PKR and T-nc886, high or medium S-nc886, and medium P-nc886 values). A second bicluster was composed also of variable ∆ct mean values (high P-nc886 and T-nc886, medium S-PKR and T-PKR, and low P-PKR and S-nc886 values). These biclusters exhibited significant different values of their composite features (Figure 4B, *p* < 2.05581 × 10^−204^ ANOVA statistics). The main differences between clusters included variables in colon tumour and colon healthy tissues (Figure 4B). The first bicluster matched with the absence of PKR in the nucleolus (Figure 4C, *p* < 0.00001, hypergeometric statistics/Fisher’s tests). The second bicluster was associated with a presence of PKR in the nucleolus (Figure 4C, *p* < 0.00005, hypergeometric statistics/Fisher’s tests). 

We independently validated the former results by calculating using ANOVA statistics with the six variables previously selected. We determined that the clusters were significantly better associated with the presence of PKR in the nucleolus (*p* < 0.000002) than the individual values (*p* < 0.035). The bicluster lacking PKR location in the nucleolus showed a relation to the positive first-line chemotherapy response (Figure 4D, *p* < 0.006, hypergeometric statistics), and the bicluster with PKR location in the nucleolus showed a relation to the negative first-line chemotherapy response (Figure 4D, *p* < 0.03, hypergeometric statistics). 

Therefore, the analysis was able to group patients whose PKR location in the cytoplasm of the tumour cells corresponded with a positive response to the treatment, and in contrast, patients with PKR restricted to the nucleolus could be grouped in clusters that corresponded with the negative response to treatment. Although the expression levels of PKR and nc886 in the different tissues analysed were necessary to determine these significant clusters, these levels were highly variable.

### 3.3. Final Outcome Was Predicted by the Expression Level of PKR and nc886 in Healthy Tissues

**Figure 5:**
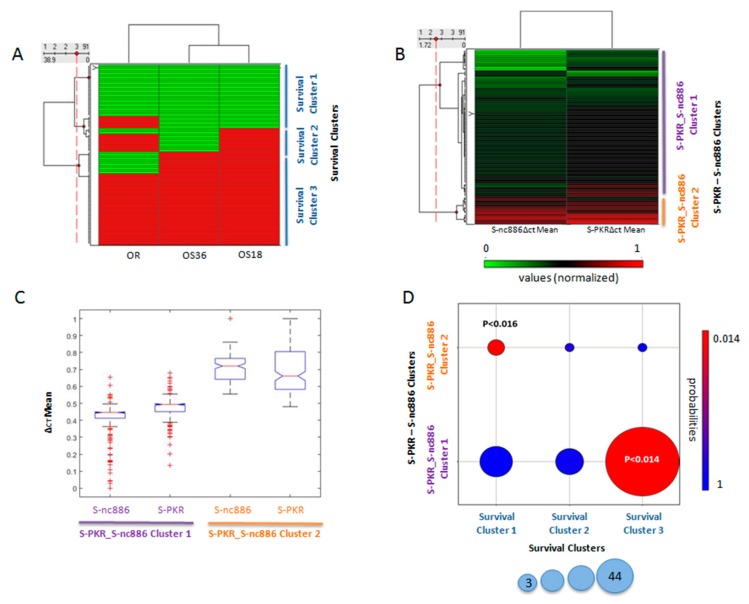
Correlation between the objective first-line chemotherapy response and the time-range patient survival with the ∆ct mean values identified by RT qPCR in colon healthy (S) tissue. (**A**) Survival clusters: Biclusters of subjects according to their objective first-line chemotherapy response, and survival after 18 and 36 months. Values are coded as follows: negative response in green and positive response in red. (**B**) S-PKR-S-nc886 clusters: Biclusters of patients according to their ∆ct mean values of nc886 and PKR in healthy tissue (S-nc886 ∆ct mean, S-PKR). ∆ct mean values are normalized between 0 (green) and 1 (red). (**C**) Boxplot of S-PKR and S-nc886 ∆ct mean values in healthy tissue for each S-PKR-S-nc886 cluster. (**D**) Co-clustering between survival clusters and the S-PKR-S-nc886 clusters. *p*-values were calculated with hypergeometric statistics. Colour code for *p*-values’ statistical significance is indicated from high (red) to low (blue). The size of the circles indicates the number of individuals in the relationship.

Finally, we raised the question about the correlation between the OR to first-line chemotherapy and the time-range patient survival after 18 and 36 months. To test the predictability effect of the measured variables, we first applied the PGMRA method to separately factorize these three measurements using NMF and we uncovered three biclusters, now called “survival clusters” (Figure 5A). Survival cluster 1 involved patients who mostly showed a negative response to the first line of treatment and died before 18 months (Figure 5A). Survival cluster 2 included patients who mostly showed a positive response to the first line of treatment and were alive after 18 months; however, they died before reaching 36 months (Figure 5A). Survival cluster 3 involved all patients who mostly showed a positive response to the first line of treatment and were alive after 18 and 36 months (Figure 5A).

On the other hand, independently, PGMRA selected the expression values of PKR and the nc886 in P, T, and S for two biclusters (Figure 5B). We found two clusters based on colon healthy tissue values from PKR and nc886, from now on called S-PKR_S-nc886 clusters. S-PKR_S-nc886 cluster 1 included a large number of patients mostly with low ∆ct mean values for nc886 and PKR. S-PKR_S-nc886 cluster 2 included fewer number of patients mostly with high ∆ct mean values for nc886 and PKR (Figure 5B). These S-PKR_S-nc886 biclusters exhibited significantly different values of their composite features (Figure 5C, *p* < 2,48689 × 10^−136^ ANOVA statistics).

Finally, we co-clustered the S-PKR_S-nc886 clusters with the survival clusters, identifying two significant associations. The most representative bicluster displayed low nc886 ∆ct mean values and medium PKR ∆ct mean values in healthy tissues (Figure 5D). This cluster was associated with a positive objective first-line chemotherapy response, and a long time-range patient survival (Figure 5D, *p* < 0.014, hypergeometric statistics/Fisher’s tests). Therefore, higher expression levels of nc886 once again selected patients with better disease outcomes. The other significant bicluster exhibited high ∆ct mean values in healthy colon tissue of the two variables PKR and nc886 (Figure 5D), and was associated with negative objective first-line chemotherapy response and short survival (Figure 5D, *p* < 0.016, hypergeometric statistics/Fisher’s tests). Therefore, lower expression levels of nc886 and PKR in healthy colon tissue selected patients with worst disease outcomes. A non-significant survival bicluster exhibiting a mixture survival values was not significantly associated with nc886 and PKR ∆ct mean values in healthy tissues (Figure 5D).

The three survival classes were independently validated using regression analysis with the clusters (rankings, see the Materials and Methods section) of healthy tissue nc886 ∆ct mean values and the healthy tissue PKR ∆ct mean values (*p* < 0.0053, F statistics).

## 4. Discussion

The identification of biomarkers that associate or predict the benefit of an appropriate selection of patient candidates for both 5-FU-based and combined therapies constitutes a broad area useful in clinical and translational research of CRC disease. However, the low specificity of chemotherapy and the great heterogeneity of the patients and samples analysed make the search for predictive biomarkers very complex [2]. Tests for microsatellite instability (MSI) and for the detection of loss of heterozygosity for chromosome 18q (18qLOH) in the early stage of the disease are beginning to be evaluated for guiding therapeutic decisions regarding the administration of 5-FU-based treatments; however, these are still under investigation. Thus, it is necessary to explore new biomarkers that can increase the portfolio to the oncologists and facilitate taking better decisions in the treatment of CRC patients. In many cancers, mutation or abnormal expression or activity of protein kinases is correlated with tumorigenesis, metastasis, and resistance to chemotherapy [39]. This study identified clusters of metastatic colon cancer patients on the basis of the kinase PKR and its modulator nc886 after the analysis of tumours and healthy samples in relation to the response to chemotherapy based on the use of the 5-FU drug (Table 1). 

In order to group patients sharing similar features within the existing heterogeneity, we approached this ambispective study using NMF techniques encoded into the PGMRA system [20]. This system was successfully used to identify complex genotypic–phenotypic architecture of mental disorders and personality traits [40], which has now been customized for cancer phenotypes. In contrast to classical clustering techniques, not all features are included in such associations, but only those that provide a multifaceted description of groups of patients at risk. These meaningful associations are termed biclusters [20,34].

When analysing the ∆ct mean values of nc886 in P and T, we identified two biclusters composed of subjects sharing ∆ct mean values of T-nc886 and P-nc886 that were associated with the OR to first-line chemotherapy. We found a significant association between patients with high levels of nc886 expression in both P and T, as well as a positive primary response to treatments based on 5-FU. Our results are consistent with previous results that show that nc886, as a Pol III transcript, is expressed abundantly and ubiquitously in all normal human tissues [41] and that its expression is increased in cancer cells [42]. In fact, most patients in our study expressed high levels of nc886 when considering the ∆ct mean values obtained in tumours (Table 1). The tumour suppressor role of nc886 has been already previously related to a better prognosis of the disease in several neoplasia such as lung, ovarian, and breast cancer, among others [17,19,43,44,45], but this study was the first time that has been related to CRC.

Moreover, we identified a smaller second significant cluster associating patients that showed a negative response to first-line treatment with lower levels of nc886 expression in the tumour. This result agrees with the previous results, where the expression of nc886 was found to be diminished or silenced in a subset of malignant cells by the DNA hypermethylation of its promoter’s CpG island [17,18,19,44,45]. Although we did not analyse the level of silencing of nc886, our results are consistent with the poor outcome detected during nc886 epigenetic repression in several neoplasms, supporting the role of tumour suppressor of nc886 also in colon cancer disease.

The levels of PKR mRNA expression could not be associated to chemotherapy response in our analysis. The Ser/Thr kinase PKR is a non-canonical kinase involved in many cellular pathways exerting various functions on cell growth and tumorigenesis [4,7,8]. Although there have been multiple studies of PKR, the exact role in cancer biology remains controversial. This is due on one hand to its ability to induce eIF2α-mediated apoptosis and on the other hand to NF-κB-mediated pro-survival effect, involved in both tumour-suppressive or oncogenic roles [6,7,8,11,14]. Because we previously identified PKR as a molecular target of 5-FU in several colon cancer cells lines [14] playing an important role in the cytotoxic effect of 5-FU, through the induction of apoptosis, in a PKR expression-dependent manner, we expected a high expression level of the PKR gene in responder patients. However, the high level of post-translational modifications and regulation of the protein indicated that PKR activity does not necessarily have to correspond to the amount of its messenger RNA. In fact, numerous proteins have been described as regulating their activity (e.g., PACT, trans-activation response (TAR) RNA binding protein (TRBP), nucleophosmin (NPM)) [4,7,8,46,47,48] and several post-translational modifications have been showed by SUMOylation and ISGylation, among others [49,50]. Nc886 has been described as a PKR inhibitor, being the inhibition of PKR/NF-kB in correlation with its tumour suppressor activity. However, recently researchers have demonstrated that nc886 can adopt two structurally distinct conformers that are functionally opposing regulators of PKR that have a second conformation able to activate PKR [12]. Therefore, whether a high level of nc886 is related to a best response to 5-FU treatment that corresponds with a high ability of PKR to induce apoptosis is still unknown and needs further investigation. In addition, a different location of PKR in the nucleus and nucleolus has been demonstrated with different forms that also suggest differences in its activity [51,52]. It has been described that PKR localizes in the cytoplasm, strongly in the nucleolus, and diffusely throughout the nucleoplasm [52]. Our analysis of total PKR expression by immunohistochemistry in 76 samples of colon tumour and their respective healthy colon tissues showed expression in the cytoplasm in all healthy tissues and in most of the colon tumour analysed. However, a smaller group of tumours were shown diffuse staining in the nucleus and, above all, staining was restricted exclusively to the nucleolus. The PGMRA analysis considering the presence or absence of PKR in the nucleolus found a bicluster where patients with PKR located in tumour cytoplasm (and absent in the nucleolus) were related with OR to first-line chemotherapy. A cluster included patients whose PKR location in the cytoplasm of the tumour cells corresponded with a positive response to the treatment, and although the levels of expression of PKR and nc886 analysed were variable between samples, they were necessary to establish statistically significant clusters. In contrast, patients with PKR restricted to the nucleolus in the tumour could be grouped significantly in a cluster that corresponded with the negative response to treatment. Therefore, an increase in the number of samples to be analysed would be convenient in future research to specifically relate the levels of nc886 with the location of PKR and the response to treatment. Although the role that PKR activity may have in the nucleus/nucleolus is not yet known, it has been suggested that PKR exists in leukaemia cell lines and patient samples in diverse molecular weight forms in the nucleus as result of several post-translational modifications. Although cytoplasmic location was detected in leukaemia low-risk patients, nuclear location was restricted to high-risk patients [51]. In addition, intrahepatic PKR nucleolar labelling was observed in human blood PBMCs and liver biopsies, with a suggested ribosome biogenesis role [53].

Finally, we found different survival clusters in those patients for which information was available (Table 1), allowing for the grouping of patients according to the expression of nc886 and PKR in healthy colon tissue. Interestingly we found two clusters significantly related to the outcome of the patients; the most representative cluster included patients with higher expression levels of nc886 and medium expression levels of PKR in the healthy colon tissues who were alive after 3 years of the first-line treatment. In contrast, lower levels of expression of both PKR and nc886 in healthy colon tissue were related with patients who died at a year and a half after the first-line treatment. Although PKR levels did not appear to be related to their activity, as we have previously discussed, for tumour cells, they interestingly remained average in healthy tissue in the cluster of patients with the best outcome, and showed less expression in healthy tissue for the cluster with worse outcome. The role of PKR as a cellular stress response protein is widely known, as PKR intervenes against numerous and varied infections, also eliminating damaged cells inducing apoptosis. In addition, PKR is able to allow the cells to live via NF-kB activation once the stress has been resolved on time [4,7,8,13]. Moreover, PKR also regulates some tumour suppressors and protein kinases involved in cancer pathways such as the signal transducers and activators of transcription factors (STATs), activating transcription factors (ATFs), tumour suppressor p53 (Tp53), the phosphatase and tensin homologue tumour suppressor (PTEN), the mitogen-activated protein kinases (MAPKs), and the toll-like receptors (TLRs), among others [4,7,8,13]. All these data suggest how important it is that PKR is expressed at adequate levels in normal tissue where it would be slightly regulated, as well as the importance that PKR would have in tumours where, regardless of its expression, its regulation can be critical.

## 5. Conclusions

In summary, although it would be convenient to increase the “n”, especially for studies where we have had less available data, we can consider that PGMA is a useful system for working with heterogeneous diseases such as cancer. PGMA analysis allowed us to identify clusters where the levels of expression of nc886 can be suggested as a potential biomarker for both the first-line response to chemotherapy and the survival of patients for at least 18 or 36 months. The higher levels of expression of nc886 in tumours, plasma, and healthy tissues were found in those patients with a better outcome. Although it is necessary to analyse a greater number of subjects to know the role of PKR as a biomarker, our data suggest that its location in the tumour cell compartments, but not its mRNA expression level, could predict the response to treatment based on the use of 5-FU in metastatic colon cancer patients.

## Figures and Tables

**Table 1:** Clusters of patients associated with objective first-line chemotherapy response and with overall survival at 18 and 36 months.

			**Sex**							
**Metastatic Colon Cancer Patients**		**Total**	**Male**	**Female**			**Age (Mean Years), SD**			
		197	127	70			65.1 ± 10.5			
**Firs-line Chemotherapy Response clusters (OR)**		**Total**	**Responders(+)**		**Non-Responders(-)**		**∆ct mean values, SD**			
		197	128		69					
P-nc886_S-nc886							**T-nc886**		**P-nc886**	
	Cluster 1	160	110		50		0.427 ± 0.09		0.289 ± 0.05	
	Cluster 2	37	18		19		0.567 ± 0.17		0.495 ± 0.14	
S-PKR_S-nc886							**S-nc886**		**S-PKR**	
	Cluster 1	77	45		32		0.386 ± 0.12		0.465 ± 0.09	
	Cluster 2	14	6		8		0.682 ± 0.09		0.703 ± 0.16	
**Survival clusters (OS)**			**First-line Response**		**OS 18 m**		**OS 36 m**			
Survival			**+**	**-**	**Survival**	**Exitus**	**Survival**	**Exitus**		
	Cluster 1	26	0	26	0	26	0	26		
	Cluster 2	18	15	3	12	6	0	18		
	Cluster 3	47	36	11	47	0	47	0		

## Supplementary Materials

The following are available online at https://www.mdpi.com/2072-6694/12/2/379/s1: Figure S1: Excel document with data. Figure S2: Study of digital dPCR expression. Figure S3: Study of EIF2AK2 expression by dPCR and qPCR. Figure S4: Study of endogenous selection in qPCR.
