# Supplementary material for: Uncovering Tumour Heterogeneity through PKR and nc886 Analysis in Metastatic Colon Cancer Patients Treated with 5-FU-Based Chemotherapy
Source: Cancers (Basel). 2020 Feb 7;12(2):379. doi: 10.3390/cancers12020379 (PMC7072376; doi:10.3390/cancers12020379)
Supplement: Supplementary file 1 [file cancers-12-00379-s001.zip › Supplementary Material/Supplementary Figure Legends.docx]

**Supplementary Figure Legends**

Figure S1: Excel Data

Figure S2. Study of dPCR expression in blood (A) and plasma (B) samples in VTRNA2 (EIF2AK2 study not shown).

Figure S3. Study of EIF2AK2 expression in dPCR and qPCR in E001 sample (plasma and tissue samples). A.1) Expression of EIF2AK2 and B2M in a plasma sample with dPCR technique. A.2) Expression of EIF2AK2 and B2M in a tissue sample with dPCR technique. B) Expression of EIF2AK2 and B2M as endogenous in plasma, non-tumoral and tumoral samples with qPCR.

Figure S4. Study of endogenous selection in qPCR. A) GAPDH endogenous test in plasma. B) HPRt endogenous test in plasma. C) B2M endogenous test in plasma.
