# Supplementary figures and images for: Uncovering Tumour Heterogeneity through PKR and nc886 Analysis in Metastatic Colon Cancer Patients Treated with 5-FU-Based Chemotherapy
Source: Cancers (Basel). 2020 Feb 7;12(2):379. doi: 10.3390/cancers12020379 (PMC7072376; doi:10.3390/cancers12020379)

A)

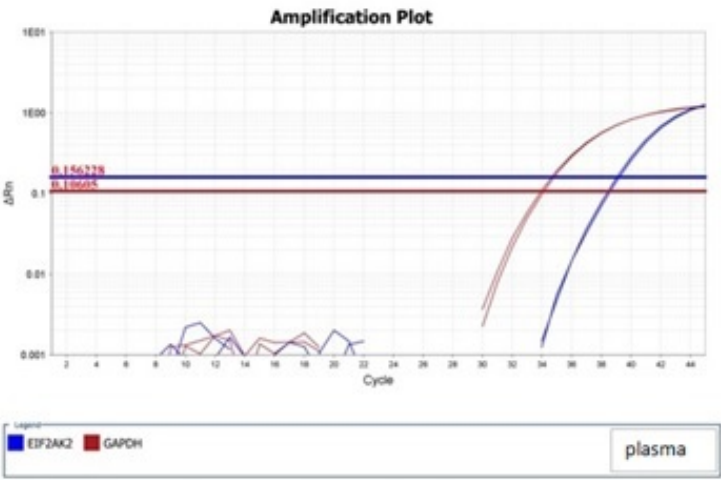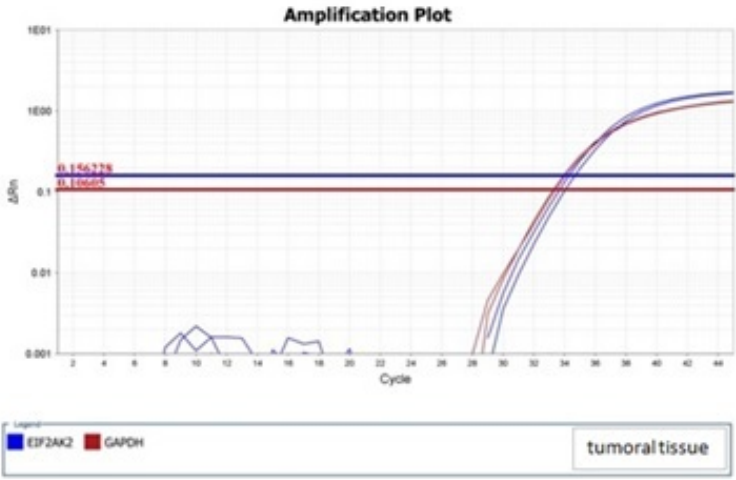

B)

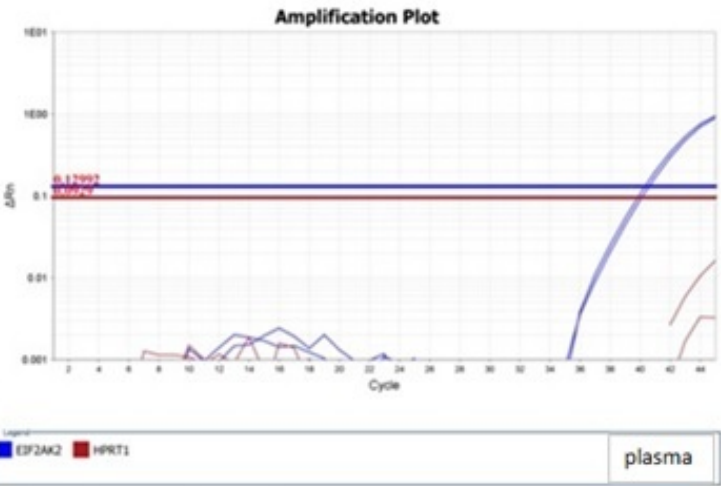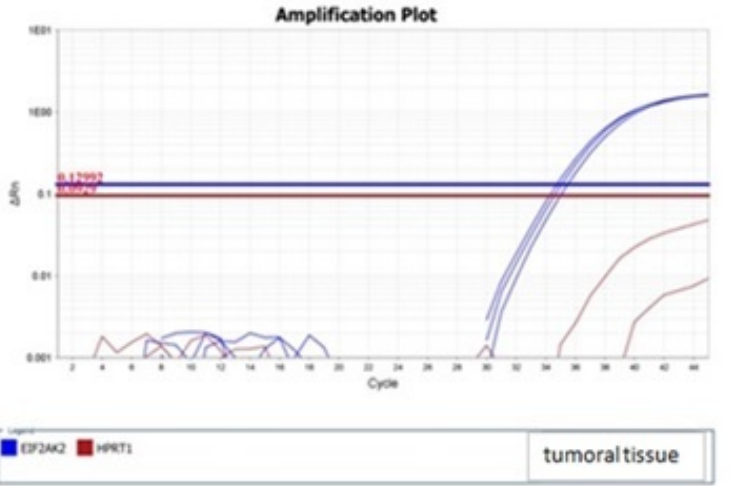

C)

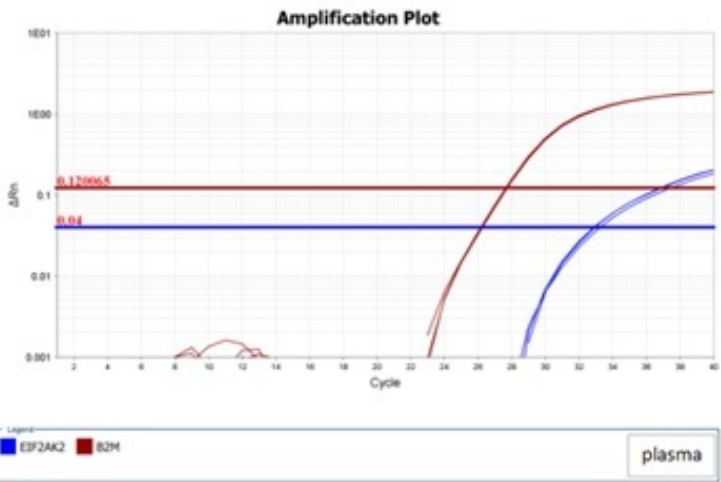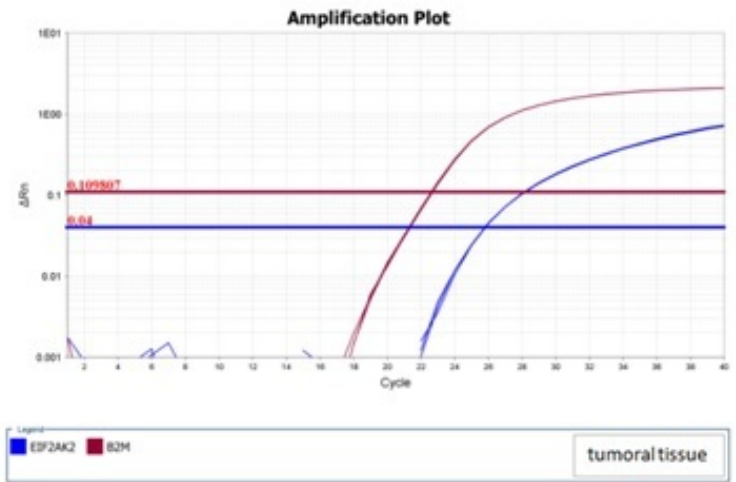

Supplement: Supplementary file 1 [file cancers-12-00379-s001.zip › Supplementary Material/Supplementary figure 4.pdf]

A)

FAM: 3 VIC: 11772 FAM+VIC: 4149 UNDETERMINED: 74 NO-AMP: 59

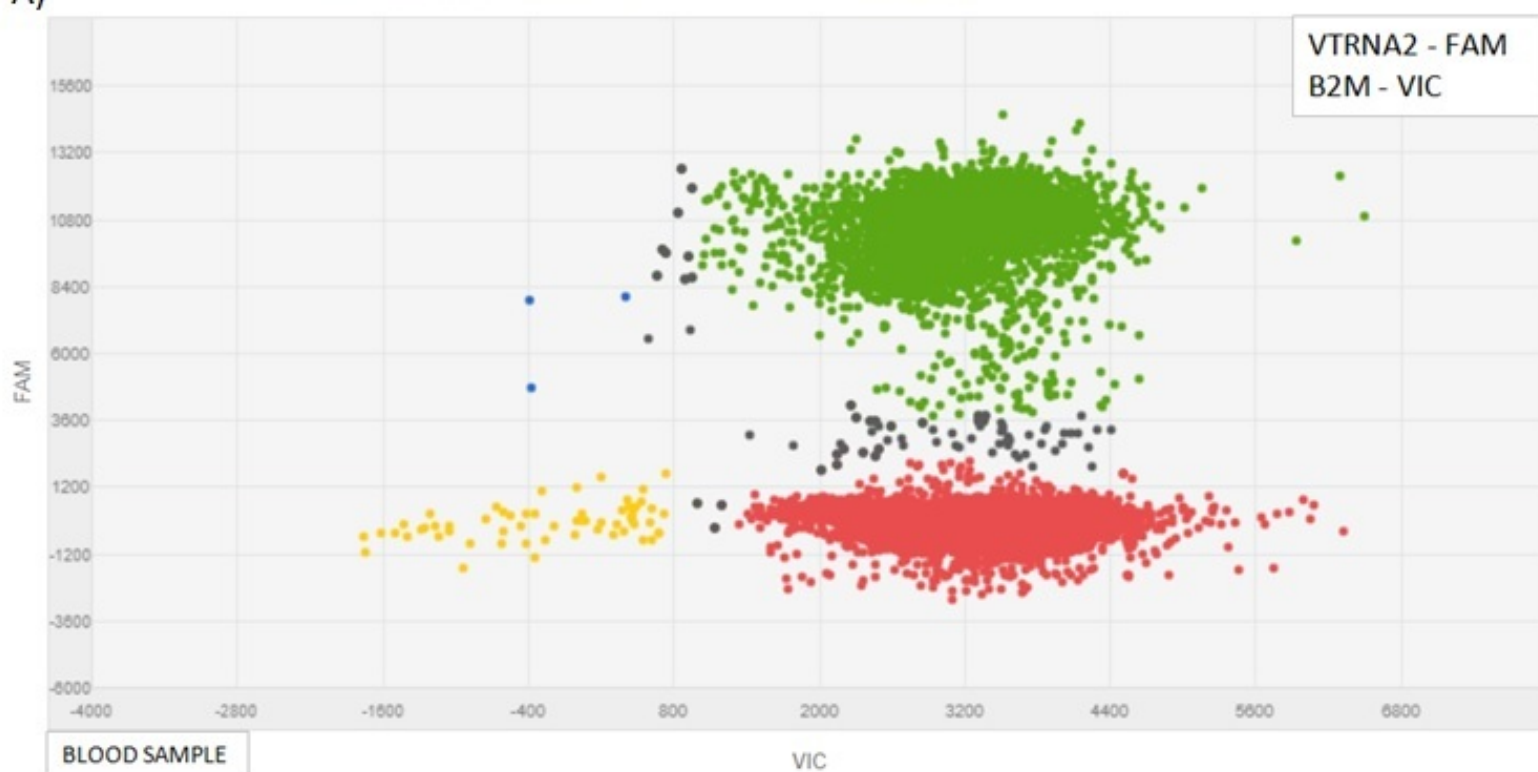

B)

FAM: 4 VIC: 433 FAM+VIC: 0 UNDETERMINED: 71 NO-AMP: 15930

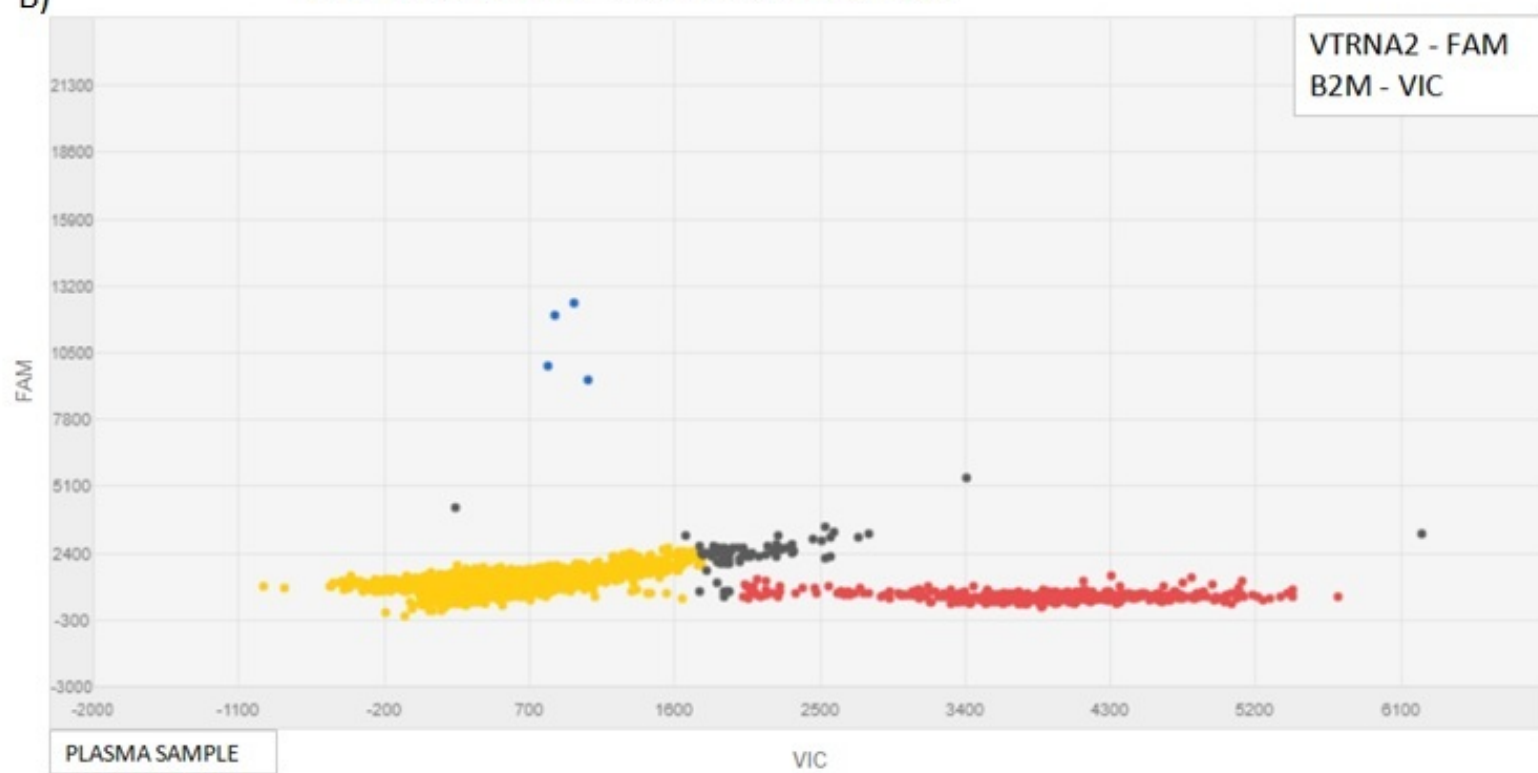

Supplement: Supplementary file 1 [file cancers-12-00379-s001.zip › Supplementary Material/Supplementary figure 2.pdf]

A.1)

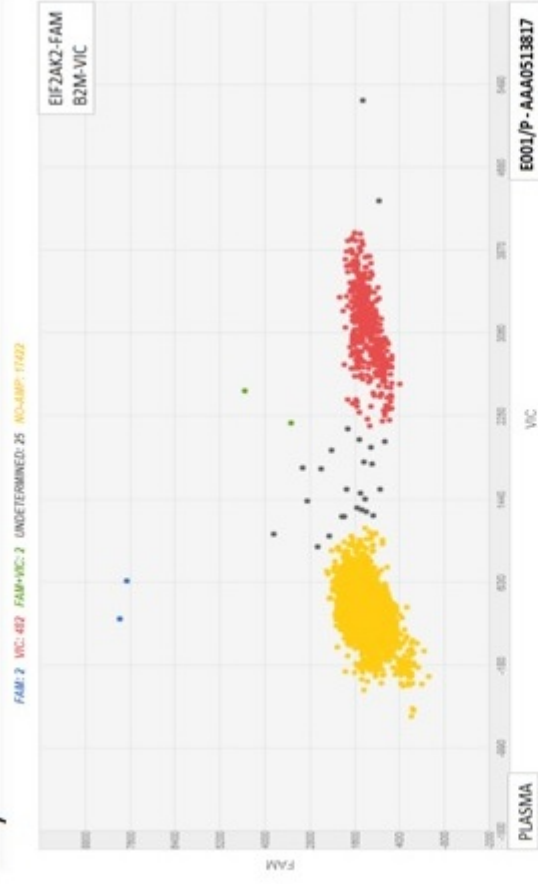

A.2)

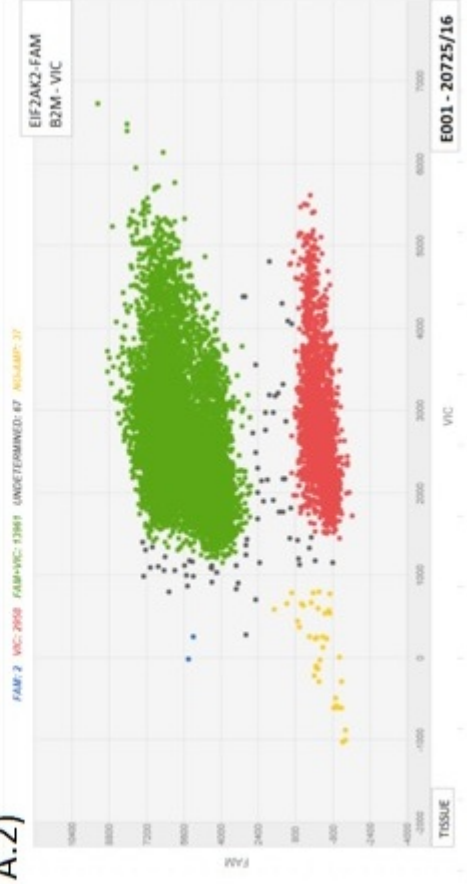

B)

RQ vs Sample

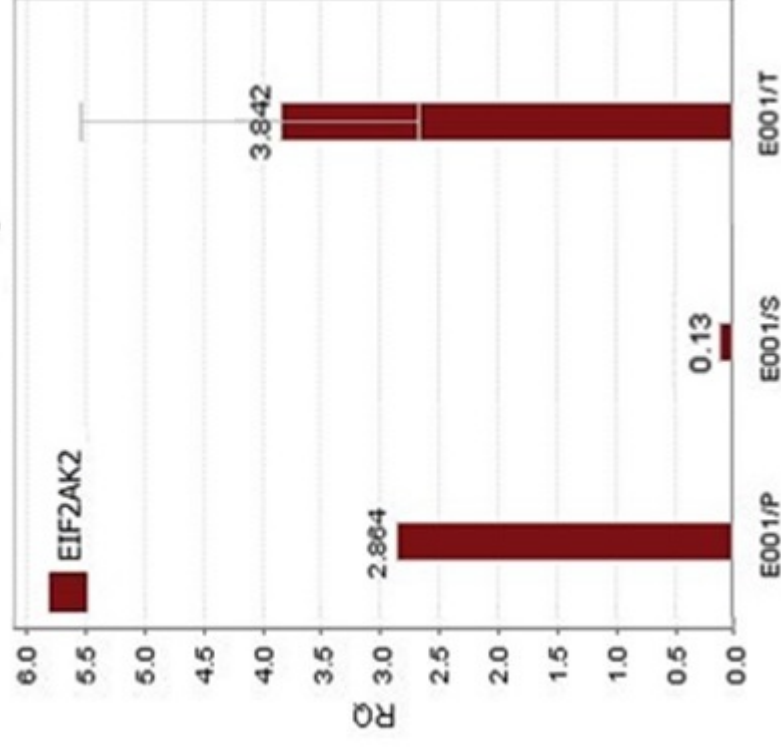

Supplement: Supplementary file 1 [file cancers-12-00379-s001.zip › Supplementary Material/Supplementary figure 3.pdf]
